# COVID-19 with social distancing, isolation, quarantine and cooperation, collaboration, coordination of care but with disproportionate impacts

**DOI:** 10.4102/safp.v62i1.5204

**Published:** 2020-09-02

**Authors:** Indiran Govender

**Affiliations:** 1 Department of Family Medicine, Faculty of Health Science, University of Pretoria and Kalafong Hospital, Pretoria, South Africa

The coronavirus disease (COVID-19) pandemic has influenced us in several ways, firstly the way we interact with one another, secondly our support for one another, thirdly how healthcare is delivered; cooperation, coordination and collaboration have become essential in making interdisciplinary, multidisciplinary and multiprofessional teamwork and collaboration between public and private healthcare obvious. Given the potential for the spread of COVID-19, we saw worldwide changes in travel policies, trade policies, supply chains and healthcare practices.

At the hospital I work in and the health district that drains into the hospital, all specialities are working together to manage the epidemic in these uncertain times. All patients coming into the hospital are screened and if necessary are tested for COVID-19. Healthcare workers from the clinics and mobile testing teams are trained in infection control measures and the use of personal protective equipment (PPE). Family Physicians are at the forefront of this epidemic being the most highly trained professionals in the district. However, doctors from all other departments join every day to assist.

It is becoming increasingly apparent that COVID-19 is not an equal-opportunity killer. In New York City, Latino and black people have been twice as likely to die from COVID-19 as white people have. The disease has been concentrated in poorer geographic settings, where people live in overcrowded areas and cannot work from home or escape to second homes. Social inequalities manifest clearly by putting lower socio- economic classes at higher risk, thus the notion of everyone who is exposed to the disease is at the same risk of death is a myth. The unequal distribution of wealth certainly equates to unequal distribution of healthcare resources. Other pandemics such as the Black Death, small pox and influenza of 1918 have also shown us this reality. In Washington, DC, black people make up 45% of COVID-19 cases but 79% of deaths. Black people made up more than 80% of hospitalized COVID-19 patients in Georgia, and almost all COVID-19 deaths in St. Louis. In Iowa, Latinos comprise more than 20% of patients, despite being only 6% of the population. Similar trends have been seen in black and South Asian patients in the United Kingdom. COVID-19 is definitely delivering an unequal blow to people of colour. The COVID-19 pandemic will undoubtedly and exponentially increase the rate of poverty across the world causing another financial depression especially affecting those who were working as daily wage, part-time, or minimum wage workers. These impacts of COVID-19 emphasises the influences of social determinantes on health. At least two of the SAAFP national conferences had this theme for their conferences. The conditions in which people live, learn, work and play greatly contributes to their health. These conditions lead to different levels of health risks, needs and outcomes among some people in certain racial and ethnic minority groups.

During the lock down, emergency units at hospitals are attending to a different profile of patients. Motor vehicle accidents are becoming less common whilst interpersonal violence, suicide and attempted suicides had increased drastically.

At least 20 shelters have been set up in Tshwane for the homeless people. The medical needs of these patients are being managed by teams led by family physicians. Family physicians have set up WhatsApp groups, webinars, zoom meetings and other COVID-19 support and resource-sharing tools to work together to manage geographic areas under their responsibility. People from different religions have come together to help with PPE, food for the homeless; students manage a call centre where community members can call for advice. It is exciting to note that at one large shelter four different religious groups – Hindus, Jews, Muslims and Christians – have united to provide a feeding programme for the homeless people in this shelter.

Since the start of the lockdown, the different political parties have worked together with a goal to fight the COVID-19 epidemic, however self-interest of politicians and political parties limit long-term cooperation. Stock markets have suffered, and the SA government has reduced interest rates to try to help the economy.

We have learned to have meetings, learn and teach using web-based apps such as Zoom, Google meetings and Microsoft teams. Blended and hybrid learning will become more prominent because of COVID-19.

Social distancing is a term, which seems to suggest that we should be socially disconnected, however, perhaps we should think more along the lines of physical distancing, since we need social and emotional connections and support now more than ever. As lockdown proceeds tensions increase in homes due to the unusual situation of having people that are not used to being together for extended periods. This in turn leads to interpersonal violence, depression and suicides. Social harmony and cohesiveness is becoming far more necessary.

This pandemic is evolving daily and doctors should use reputable information sources, such as World Health Organization, National Institute for Communicable Diseases and Centre for Disease Control and Prevention since with more online use there has been an increase in fake news.

We are collectively in a situation in which we have no experience and for which we have limited preparation. My thoughts are that this disaster will teach us that cooperation, collaboration and coordination is essential for healthcare and especially for universal healthcare in South Africa. The relationships and coordination of care we have built must continue post COVID-19. This pandemic like other pandemics shows us that although everyone is at risk for the disease, the prognosis is definitely dependant on where we stand in terms of social class.

